# Inherited Paediatric Motor Neuron Disorders: Beyond Spinal Muscular Atrophy

**DOI:** 10.1155/2017/6509493

**Published:** 2017-05-28

**Authors:** Hooi Ling Teoh, Kate Carey, Hugo Sampaio, David Mowat, Tony Roscioli, Michelle Farrar

**Affiliations:** ^1^Department of Paediatric Neurology, Sydney Children's Hospital, Randwick, NSW, Australia; ^2^Discipline of Paediatrics, School of Women's and Children's Health, UNSW Medicine, UNSW Sydney, Randwick, NSW, Australia; ^3^Centre for Clinical Genetics, Sydney Children's Hospital, Randwick, NSW, Australia; ^4^St Vincent's Clinical School, UNSW Medicine, UNSW Sydney, Darlinghurst, NSW, Australia

## Abstract

Paediatric motor neuron diseases encompass a group of neurodegenerative diseases characterised by the onset of muscle weakness and atrophy before the age of 18 years, attributable to motor neuron loss across various neuronal networks in the brain and spinal cord. While the genetic underpinnings are diverse, advances in next generation sequencing have transformed diagnostic paradigms. This has reinforced the clinical phenotyping and molecular genetic expertise required to navigate the complexities of such diagnoses. In turn, improved genetic technology and subsequent gene identification have enabled further insights into the mechanisms of motor neuron degeneration and how these diseases form part of a neurodegenerative disorder spectrum. Common pathophysiologies include abnormalities in axonal architecture and function, RNA processing, and protein quality control. This review incorporates an overview of the clinical manifestations, genetics, and pathophysiology of inherited paediatric motor neuron disorders beyond classic SMN1-related spinal muscular atrophy and describes recent advances in next generation sequencing and its clinical application. Specific disease-modifying treatment is becoming a clinical reality in some disorders of the motor neuron highlighting the importance of a timely and specific diagnosis.

## 1. Introduction

Motor neuron diseases (MND) are a heterogeneous group of progressive disorders resulting in difficulties walking, moving, breathing, and swallowing, with onset ranging from birth to adulthood. The hallmarks are weakness and atrophy from disruption of the lower motor neuron and/or spasticity from upper motor neuron dysfunction. Globally, the most common motor neuronopathy in children is attributable to picornavirus infections, including coxsackievirus, enterovirus 68 and 71, echovirus, and polio, presenting with an acute flaccid paralysis that may occur in outbreaks [1, 2]. While a variety of causes may be considered in children presenting with a motor neuron syndrome (hereditary, immune mediated, infectious, paraneoplastic, sporadic, or toxic), in those with insidious onset and slow progression, a genetic basis is frequently considered. The number of genetic causes identified which show variability and overlap in clinical phenotype continue to expand in the era of genomic testing. In children, the most common inherited motor neuron disease is spinal muscular atrophy (SMA) due to homozygous disruption of the survival motor neuron 1 (*SMN1*) gene, with classic phenotypes prompting targeted SMN1 gene testing [3]. Beyond typical SMA and predominant lower motor neuron phenotypes, pathology may be more extensive including numerous genes associated with MNDs in children and young people.

While paediatric motor neuron diseases may have clinical manifestations confined to anterior horn cell disease, less common “SMA plus” or atypical SMA phenotypes, may also demonstrate additional features indicative of more extensive neurologic or multisystem involvement, including deafness, epilepsy, encephalopathy, spasticity, visual impairment, and brainstem, cerebellar, gastrointestinal, or rheumatological disorders [4, 5]. Alternatively, amyotrophy may develop later or may indeed not be the major presentation in multisystem disorders. Furthermore, primary involvement of the upper motor neuron, termed juvenile amyotrophic lateral sclerosis (jALS), may be evident. The many causative genes are ubiquitously expressed and provide clinical, pathological, and genetic insights into paediatric motor neuron degeneration as a spectrum of neurodegenerative disorders.

Next generation sequencing (NGS), comprising gene panels, whole exome sequencing (WES), and whole genome sequencing (WGS), has revolutionised the paradigm of clinical genetic testing for rare diseases. “Neurogenomics,” a new era of genetics in neurology, critically relies on careful clinical phenotyping to accurately interpret molecular genetic results. Here, we review paediatric motor neuron diseases beyond SMA and present their distinguishing clinical and neuropathologic features together with an analysis of causative genes and the disease mechanisms underlying motor neuron degeneration. Specific disease-modifying treatment is evaluated when available, highlighting the importance of a specific genetic diagnosis. We have included “SMA plus” syndromes in which motor neuron dysfunction is the primary but not the sole feature, juvenile amyotrophic lateral sclerosis (jALS), and multisystem disorders in which amyotrophy related to lower motor neuron degeneration is evident. Hereditary spastic paraplegias and pure distal hereditary motor neuropathies have been extensively reviewed and are therefore not described [6–8]. In addition, we also provide an overview of NGS technologies that may be utilised in molecular genetic diagnostic approaches. These are currently transforming clinical practice and may provide a method to establish a timely cost-effective diagnosis, critical to enabling therapy at the earliest possible stage for these devastating groups of disorders. The importance of a multidisciplinary approach to navigate the complexities of molecular genetic diagnosis is highlighted.

## 2. Clinical Presentations, Molecular Genetics, and Specific Disease-Modifying Therapy

**Figure 1:**
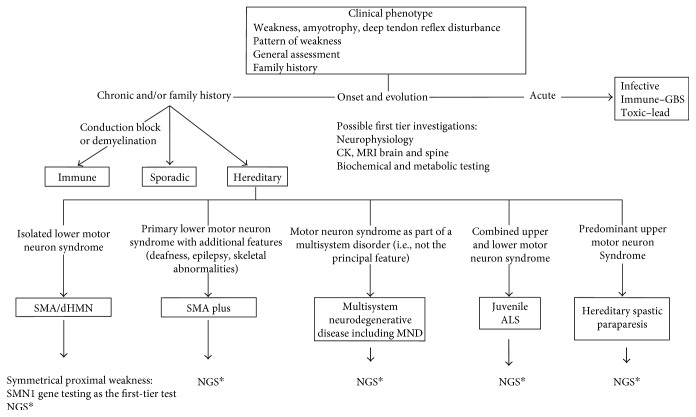
Diagnostic approach for a patient (<18 years) with a motor neuron disorder (MND). ALS: amyotrophic lateral sclerosis; CK: creatinine kinase; dHMN: distal hereditary motor neuropathy; GBS: Guillain-Barre syndrome; MND: motor neuron disorder; MRI: magnetic resonance imaging; NGS: next generation sequencing. ^∗^genomic testing by panel; WES or WGS may be an option depending on local availability.

The clinical approach to a paediatric patient presenting with a motor neuron syndrome includes assessment of the pattern of weakness, amyotrophy and progression (proximal or distal, bulbar or respiratory involvement), presence of spasticity and deep tendon reflexes, and family history. Conventional electrophysiological studies and electromyography (EMG) are important in establishing a neurogenic basis and differentiating from myopathy and neuromuscular junction disorders. In lower motor neuron disorders, compound muscle action potentials (CMAPs) are reduced with normal or slightly slowed motor conduction velocities consistent with axonal loss. EMG may demonstrate active denervation with positive sharp waves and fibrillation potentials and/or high-amplitude polyphasic motor units of long duration, suggestive of reinnervation. Assessments of vision, hearing, cognition/development, and general systems are important in obtaining a detailed phenotype. Neuroimaging of the brain and spine and ophthalmological and audiological studies may complement clinical assessment. Second-tier testing may include muscle or nerve biopsy, metabolic tests, or single gene testing based on phenotype. NGS technologies are transforming clinical practice and increasingly being used as a first-tier diagnostic test to provide a timely diagnosis (Figure 1). This is reinforcing the importance of accurate clinical evaluation together with knowledge of the diverse clinical and genetic phenotypes to be able to provide molecular genetic diagnoses.

## 3. SMA Plus Syndromes

SMA plus syndromes, or atypical SMA, encompass disorders in which lower motor neuron dysfunction is the primary but not the sole feature and may be classified by distinct patterns of muscle weakness (summarised in Table 1). SMA with pontocerebellar hypoplasia (SMAPCH1/PCH1), SMA with progressive myoclonic epilepsy (SMAPME), congenital SMA with arthrogryposis and fractures, and SMA caused by mitochondrial disorders display predominant proximal weakness. SMA with lower extremity dominance (SMALED) and scapuloperoneal SMA have distinct patterns of weakness as described. Brown-Vialetto-Van Laere (BVVL) syndrome and related disorders have prominent bulbar weakness.

### 3.1. Pontocerebellar Hypoplasia with Spinal Muscular Atrophy (PCH1)

Pontocerebellar hypoplasia (PCH) refers to a group of severe neurodegenerative disorders characterised by spinocerebellar degeneration, malformation of the vermis and anterior lobe of the cerebellum, and severe hypoplasia of the brainstem. Ten subtypes of PCH have been recognised based on clinical and pathological manifestations, with PCH type 1 associated with anterior horn cell disease, resembling infantile spinal muscular atrophy [9–12]. Mutations segregating in an autosomal recessive way in the vaccinia-related kinase 1 (*VRK1*), exosome component 3 (*EXOCS3*), and exosome component 8 (*EXOCS8*) genes are causative in PCH type 1A, type 1B, and type 1C, respectively. The majority of patients with PCH1 (between 30 and 75 percent) have pathogenic variants in *EXOCS3*, with mutations in *VRK1* and *EXOCS8* confined to several case reports only [12–15]. The genetic aetiology remains to be elucidated in additional PCH1 patients and NGS may provide opportunities to further understand pathogenesis.

*TSEN54* mutations account for >90% of described alleles associated with PCH2 and PCH4, however, has also been described in a single case with PCH1 [16]. The latter was expressed in the brainstem and cerebellum but not in anterior horn cells and confirmation in other families is awaited. In 2016, a further two SMAPCH genes were identified using WES. These included mutations in the *SLC254A6* profission mitochondrial protein reported in two families with lethal pontocerebellar hypoplasia with optic atrophy and sensorimotor neuropathy [17] and a de novo *MORC2* mutation in a single patient [18].

The clinical characteristics associated with “classical” PCH1 are severe hypotonia, areflexia, muscle weakness, central visual impairment, dysphagia, respiratory insufficiency, and acquired microcephaly with clinical onset in the first months of life and death in early infancy. Ataxia, nystagmus, and psychomotor retardation may be additional features. Antenatal onset, with congenital contractures and polyhydramnios, in severe phenotypes and later onset in milder phenotypes, with survival beyond adulthood, have also been described [11, 15]. MRI brain shows cerebellar hypoplasia, with involvement of the cerebellar hemispheres, together with variable involvement of the pons and cerebrum. Generalised cortical atrophy may be seen in longer-surviving patients. Electrophysiology demonstrates a sensory-motor axonal neuropathy and acute denervation on electromyography (EMG).

There are genotype-phenotype correlations within *EXOCS3* pathogenic variants related to clinical outcome, survival, and extent of pontine hypoplasia. Patients with homozygous p.D132A missense mutations have a milder phenotype, better preservation of the pons, and survival [14]. Patients with homozygous p.G135E mutations or altered reading frame mutations demonstrate severe phenotypes and death in childhood.

PCH1A and PCH1B show distinct clinical manifestations with variability. Severe congenital microcephaly, early-onset SMA, mild or moderate intellectual disability, and survival to late childhood are characteristics of PCH1A [15]. The PCH1A phenotype has broadened to include peripheral neuropathy, microcephaly with simplified gyral pattern, spasticity, and normal cognitive function [19, 20]. In contrast, the PCH1B phenotype usually includes severe intellectual disability with minimal or no language acquisition, acquired and progressive microcephaly, oculomotor dysfunction, and lack of fixation [12]. Rare early onset of complicated spastic paraplegia with mild pontocerebellar hypoplasia has been recognised as a variant syndrome of PCH1B [21], providing clinical insights into more extensive motor network dysfunction. The PCH1C phenotype encompasses severe muscle weakness and failure to thrive with delayed psychomotor development, often with visual and hearing impairment and respiratory problems causing early death. MRI brain shows features of cerebellar hypoplasia, corpus callosum hypoplasia, and hypomyelination [13].

*VRK1* encodes a serine/threonine kinase, associated with cell cycle regulation, histone modification, DNA repair responses, and disruption of RNA processing, which are common pathophysiologic themes underlying motor neuron diseases [22]. Similarly, impaired mRNA metabolism due to exosome dysfunction underlies PCH1B and C, with *EXOSC3* and *EXOSC8* encoding core components of the RNA exosomes that process and degrade RNA, regulating activity of gene expression. However, while EXOSC3 mutations affect mostly spinal motor neurons and Purkinje cells, oligodendroglia cells are also targets of mutations in EXOSC8 [13].

To date, no disease-modifying therapy for SMAPCH1 has been established, and management is symptomatic including nutritional and respiratory support as indicated.

### 3.2. Spinal Muscular Atrophy with Progressive Myoclonic Epilepsy (SMAPME)

**Figure 2:**
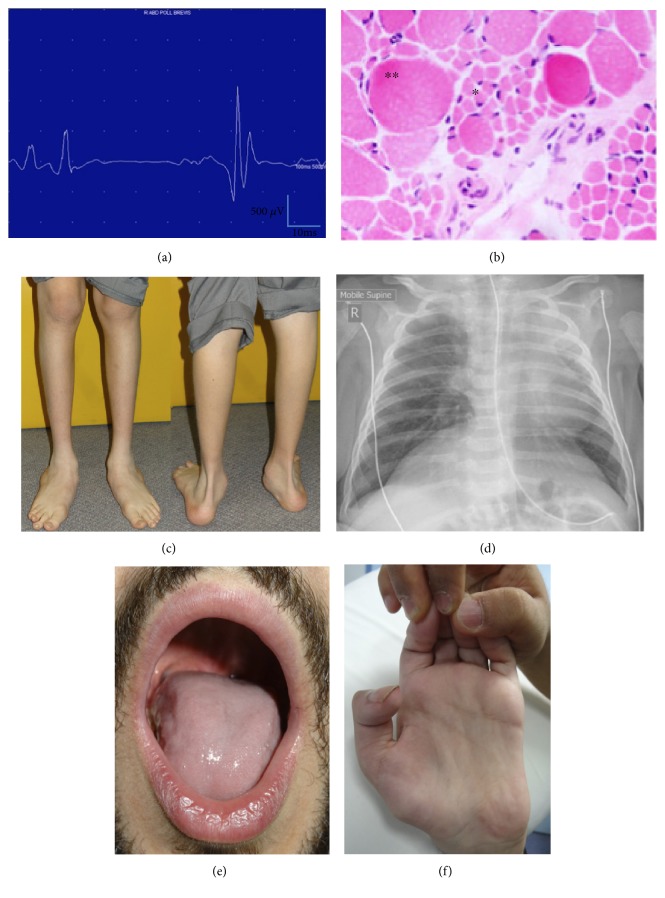
Clinical pictures of SMA plus syndrome. (a) EMG demonstrating a large-amplitude polyphasic motor unit characteristic of reinnervation. (b) H&E magnification ×200 showing small round denervated muscle fibers^∗^ and large hypertrophic muscle fibers^∗∗^. (c) Distal leg atrophy. (d) CXR showing diaphragm eventration in spinal muscular atrophy with respiratory distress (SMARD. (e) Tongue atrophy seen in Brown-Vialetto-Van Laere syndrome. (f) Hand wasting with contractures seen in Brown-Vialetto-Van Laere syndrome.

SMAPME was first reported by Jankovic and Rivera in 1979 [23]. It is characterised by childhood onset progressive proximal muscle weakness, hypotonia, areflexia, and muscle wasting after initial normal developmental milestones [24]. Mild facial weakness, tongue fasciculations, sensorineural hearing loss, and tremor may be present. Restrictive respiratory disease and recurrent infections occur with progressive weakness and dysphagia. Treatment resistant myoclonic epilepsy occurs later in the course of disease with survival limited to the first two decades due to respiratory insufficiency. Rare phenotypic variants described recently include polyarticular arthritis and spinal muscular atrophy, mild SMA phenotype with no myoclonic epilepsy, eyelid myoclonic status epilepticus, and later presentations in adolescence with absence epilepsy and atonic seizures [25–28]. MRI brain is normal. EMG demonstrates active denervation with positive sharp waves and fibrillation potentials together with large amplitude, long duration, and polyphasic motor units, consistent with reinnervation (Figure 2(a)). Electroencephalograph (EEG) may be normal or may demonstrate a slow background with epileptic activity that may be photosensitive. Epileptic activity may clinically correspond to limb jerks, head nodding, or negative myoclonus [24, 29].

Mutations in N-acylsphingosine amidohydrolase 1 (*ASAH1*), encoding the lysosomal enzyme acid ceramidase, were identified by WES as the basis for an autosomal recessive form of SMAPME [24]. Interestingly, mutations in *ASAH1* are also associated with Farber lipogranulomatosis, a severe early-onset lysosomal storage disorder affecting multiple tissues characterised by the accumulation of sphingolipids. In vitro acid ceramidase levels are <10% in Farber disease, compared to <30% in SMAPME, which correlated with phenotypic variability. Zhou et al. [24] demonstrated the functional impact of ASAH1 missense mutations in vivo, with reduction in acid ceramidase activity causing marked defects of motor-axonal branching and a significant increase in apoptosis in the spinal cord. Furthermore, ceramides are precursors of complex sphingolipids, which are important for normal functioning of both the developing and the mature brain and myelin.

There is currently no disease-modifying treatment available for SMAPME. Myoclonic epilepsy can be treated with antiepileptics but is generally resistant to conventional therapy. Haematopoietic bone marrow transplantation has been used in other lysosomal diseases and Farber's disease [30]. Like many other lysosomal storage disorders with severe CNS disease, haematopoietic stem cell transplantation has been disappointing in ameliorating neurological manifestations. Allogenic stem cell transplantation has successfully almost completely resolved granulomas in children with Farber's disease, with predominant systemic disease and no neurological involvement, producing improvement in mobility and joint contractures [31, 32].

### 3.3. SMA with Congenital Arthrogryposis and Fractures

The clinical features of infantile SMA with arthrogryposis reflect antenatal onset of weakness, accompanied by disturbances in bone and cardiac development. This includes a history of reduced fetal movements, polyhydramnios, breech presentation, pulmonary hypoplasia with diaphragmatic eventration, and early death. Arthrogryposis, osteopenia, multiple fractures, and congenital cardiac defects are typically present at birth. Severe hypotonia, muscle weakness, areflexia, and tongue fasciculations are present. Respiratory difficulty, kyphosis, scoliosis, mild micrognathia, and reduced facial expression result from muscle weakness. Electrophysiology and muscle pathology confirm denervation (Figure 2(b)). Most children do not survive greater than several months due to respiratory insufficiency without extensive respiratory and medical support. To date, no disease-modifying therapy for congenital SMA with arthrogryposis or fractures has been recognised.

Recessive mutations in four distinct genes have been identified in congenital/infantile SMA with arthrogryposis or fractures, including survival motor neuron 1 (*SMN1*) [33], thyroid hormone receptor interactor 4 (*TRIP4*), activating signal cointegrator 1 complex subunit 1 (*ASCC1*) [34], and ubiquitin-like modifier-activating enzyme 1 (*UBA1*). Together, these disorders are exceptionally rare, with autosomal recessive or X-linked recessive (*UBA1*) inheritance and descriptions confined to case reports [34–39].

### 3.4. Lethal Arthrogryposis with Anterior Horn Cell Disease (LAAHD)

LAAHD is an autosomal recessive disease described originally in 11 Finnish families associated with recessive pathogenic variants in the RNA export mediator, *GLE1* [40, 41]. Clinical features include fetal immobility with multiple distal, inward spiral joint contractures, low-set ears, hypoplastic jaw, short neck, some lung hypoplasias, mild hydrops, and fetal death in pregnancy or the early postnatal period [41]. Size and shape of the spinal cord are normal, but the anterior horn motor neurons appear degenerated and diminished in number. The cerebrum and cerebellum are not affected. *GLE1* is an evolutionarily conserved gene that encodes a protein that is required for export of mRNA from the nucleus to cytoplasm, thus regulating gene expression at multiple steps, including nuclear mRNA export, translation initiation, and translation termination.

In addition to LAAHD, GLE1 variants are recognised in ALS with autosomal dominant inheritance and autosomal recessive lethal congenital contracture syndrome 1 (LCCS1). LCCS1 has the most severe phenotype with extreme hypoplasia of skeletal muscle and thinning of the spinal cord due to paucity of anterior horn cells. This disorder has thus far only been observed in Finland and is usually fatal before 32 weeks of pregnancy, presenting with total immobility, severe hydrops, and intrauterine growth retardation [41, 42].

Lethal congenital contracture syndrome 2 (LCCS2) also includes cranial and ocular abnormalities and striking enlargement of the bladder accompanied by hydronephrosis and cystic changes of the kidneys. The majority of affected cases have been stillborn or died soon after birth due to respiratory insufficiency with post mortems confirming anterior horn cell pathology. Phenotypic variability for this syndrome has been recognised in two adolescent sisters aged 12 and 13 years with arthrogryposis, amyotrophy, facial paresis, myopia, and degenerative vitreoretinopathy [43]. Pathogenic variants in the v-erb-b2 erythroblastic leukemia viral oncogene homolog 3 (*ERBB3*) gene have been described in this consanguineous Israeli-Bedouin family with LCCS2 segregating as an autosomal recessive trait [44].

### 3.5. SMA Plus Syndromes Caused by Mitochondrial Disorders

SMA caused by mitochondrial disorders usually present with a severe infantile SMA, or Werdnig Hoffman, a disease characterised by hypotonia, weakness, and ensuing respiratory failure. Clinical manifestations are more extensive and may include infantile onset hypertrophic cardiomyopathy, hepatic failure, spasticity, a Leigh-like syndrome with encephalopathy, brainstem dysfunction, seizures and psychomotor delay, ptosis, and ophthalmoplegia [45]. Biochemical features may include lactic acidosis and elevated creatinine kinase. Muscle biopsy may show grouped atrophy, reduced cytochrome C oxidase (COX) staining, and low activity of respiratory chain enzyme complex activity.

The genes known to cause SMA phenotypes with mitochondrial disorders are *SCO2*, *TK2*, and *DGUOK*. *SCO2* encodes one of the COX assembly proteins associated with copper insertion into the holoenzyme involved in the biogenesis of COX subunit II that is important in the function of the mitochondrial inner membrane while the latter two are mitochondrial depletion syndromes, characterised by reductions in the amount of mitochondrial DNA which impairs the synthesis of respiratory chain complexes. Mitochondrial dysfunction has been implicated in many neurodegenerative conditions including motor neuron diseases in which motor neuron energy demands are met by mitochondrial oxidative phosphorylation. Distinct genotype-phenotype correlations are seen and age of onset and progression may be modulated by the pathogenicity of the specific mutation, especially in diseases caused by compound heterozygous variants. *SCO2* mutations are associated with infantile onset cardioencephalomyopathy with cerebral white matter and basal ganglia lesions, neurogenic muscular atrophy, and hypertrophic cardiomyopathy [45, 46]; *DGUOK* is also known as hepatocerebral syndrome (MTDPS3—mitochondrial DNA depletion syndrome 3) and characterised by SMA with infantile onset liver dysfunction, nystagmus, cerebral atrophy, and death from liver failure before age 12 months [47]. Interestingly, adult-onset lower motor neuron disease, progressive external ophthalmoplegia, ptosis, and limb girdle myopathy have also been reported with *DGUOK* mutations. *TK2* is associated with mitochondrial DNA depletion syndrome 2 (MTDPS2), commonly associated with myopathy with a wide clinical variability that includes intrafamilial variability where in a sibling pair, the brother had electrophysiologic findings compatible with SMA and was alive at age 4 years while his sister died at 2 years with severe weakness and hypotonia from the first months of life [48]. Specific therapy for SMA caused by mitochondrial disorders is at the moment represented by generic mitochondrial disorder therapy and aims to alter mitochondrial dynamics by enhancing respiratory chain function and eliminating toxic metabolites. Clinical response to these treatments has been observed in animal models, but translation to benefit in humans is yet to be shown. Copper supplementation for *SCO2* mutations that although does not alter brain and muscle symptoms, have shown reversal of the hypertrophic cardiomyopathy [49].

### 3.6. Spinal Muscular Atrophy with Lower Extremity Predominant (SMALED)

SMALED is typically characterised by congenital or early onset, often at or before 5 years of age, proximal > distal lower limb-predominant muscle weakness, and atrophy with normal sensation, often resulting in significant mobility impairment. Clinical features include delayed walking, waddling gait, difficulty walking, and loss of distal reflexes. Congenital arthrogryposis, fasciculations, foot deformities with calcaneovalgus, pes planus and pes valgus (Figure 2(c)), contractures, hip dysplasia, or hyperlordosis may be evident [50]. The disorder is static or mildly progressive and the degree of muscle wasting does not predict severity of weakness. Following initial clinical and pathological descriptions of anterior horn cell disease, the phenotype has broadened to include spasticity and cognitive impairment, indicative of broader motor network neuronal dysfunction in some individuals [51]. Furthermore, adult-onset presentations or upper limb involvement with scapular winging and minimal distal hand weakness are recognised [51, 52].

MRI of the upper and lower limbs demonstrates a characteristic pattern of muscle fat replacement and selective hypertrophy. In the lower limb, there was diffuse involvement of rectus femoris, vastus lateralis, vastus intermedius, tibialis anterior, and gastrocnemius, with relative sparing of medial adductors and semitendinosus and peroneal muscles. Cortical malformations, of the frontal and perisylvian regions, have been described in SMALED1 patients with intellectual disability on neuroimaging [51]. To date, no disease-modifying therapy for SMALED has been established.

NGS techniques established autosomal dominant mutations of dynein cytoplasmic 1 heavy chain 1 (*DYNC1H1*) gene [53] or motor adaptor bicaudal D homolog 2 (*BICD2*) gene [54, 55] as causes of SMALED 1 and SMALED2, respectively. These proteins are important in axonal transport and the functional disturbances identified reveal their role in motor neuron disease. *DYNC1H1* encodes the dynein heavy chain protein that forms the crucial core of the cytoplasmic dynein complex, which is essential for retrograde axonal transport, the maintenance of protein homeostasis, and neuronal migration. Likewise, the BICD2 protein interacts with the dynein-dynactin motor complex also important in axonal transport. Both motor and tail domain mutations in *DYNC1H1* have been described [53, 56]. Pathogenic variants in *DYNC1H1* may also be allelic for other neurological disorders including Charcot-Marie-Tooth disease; axonal, type 20, CMT20, and mental retardation; and autosomal dominant 13, with neuronal migration defects, MRD13. Some patients with MRD13 show clinical evidence of peripheral neuropathy [57]. More recently, *BICD2* variant syndrome includes arthrogryposis multiplex congenital with bilateral perisylvian polymicrogyria and a phenotype with chronic myopathy [58, 59].

### 3.7. Scapuloperoneal Spinal Muscular Atrophy (SPSMA)

SPSMA is characterised by neurogenic scapuloperoneal amyotrophy, congenital absence of muscles, progressive scapuloperoneal atrophy, and progressive distal weakness and amyotrophy. Additional discerning features may include laryngeal palsy and occasional sensorineural deafness, short stature, scoliosis, and mild limb and vertebral skeletal dysplasias. Recently, a phenotype combining scapuloperoneal spinal muscular atrophy and skeletal dysplasia and interfamilial phenotypic variability where the father showed a mild form of SPSMA while the son presented at birth with skeletal dysplasia and a progressive course was described, possibly suggesting a progression of the disease in generations [60, 61].

SPSMA is an autosomal dominant condition caused by mutations in the transient receptor potential vanilloid 4 (*TRPV4*) channel gene. *TRPV4* encodes a calcium permeable nonselective cation channel expressed in multiple tissues and cell types, including smooth muscle cells and the bones. In the peripheral nervous system, this gene is expressed in skin sensory receptors, in dorsal root ganglia, and, to a lesser extent, in motor neurons. Thus, mutations in *TRPV4* are allelic for a number of different disorders including congenital spinal muscular atrophy and arthrogryposis, autosomal dominant axonal Charcot-Marie-Tooth disease type 2C, distal hereditary motor neuropathy [62, 63], and a range of skeletal dysplasias [64]. The clinical and MRI features of *TRPV4*-spinal muscular atrophy, nonprogressive, lower extremity predominant are subtly different to *DYNC1H1*- and *BICD2*-SMALED; for instance, deformities are equinovarus rather than calcaneovalgus and lower limb MRI shows sparing of biceps femoris and medial gastrocnemius rather than thigh adductors and semitendinosus [65].

### 3.8. Spinal Muscular Atrophy with Respiratory Distress (SMARD)

SMARD is a rare autosomal recessive motor neuron disorder characterised by predominantly distal muscle weakness with diaphragmatic palsy resulting in severe respiratory failure resulting in premature death. Symptoms typically present early between six weeks and six months. In affected infants, intrauterine growth retardation, weak cry, and suck or congenital foot deformities are the first symptoms followed by respiratory failure due to diaphragmatic paralysis (Figure 2(d)) and progressive muscle weakness. However, high variability in presentations within a family and a late onset and slow progressive form has been reported [66, 67]. Further peripheral neuropathy with no respiratory involvement has been described [68, 69]. Other clinical features indicative of pathology beyond SMA include sensory and autonomic dysfunction (excessive sweating, urinary retention, constipation, and cardiac arrhythmia), seizures, and progression of cranial nerve paresis [70]. Histological and ultrastructural findings of sural nerve showed Wallerian degeneration and marked axonal atrophy with hypo-hypermyelination and variability in myelin thickness [71]. These changes also extend from the spinal cord in motor nerves.

SMARD1 is caused by homozygous or compound heterozygous mutations in the gene encoding immunoglobulin *μ*-binding protein 2 (*IGHMBP2*) [72]. The gene is ubiquitously expressed and encodes an ATPase/helicase responsible for the separation of double-stranded DNA and RNA. *IGHMBP2* gene consists of 15 exons and mutations are distributed along all the 15 exons of the gene but are more frequent in exons 10 and 12. However, the exact role of the protein and correlation between the type of mutation and the phenotype remain unclear [70]. The clinical phenotype differences and outcome could be related to the IGHMBP2 protein levels. A single case with similar phenotype caused by an X-linked recessive mutation in the ribosomal biogenesis protein *LAS1L* is designated SMARD2 [73].

### 3.9. Brown-Vialetto-Van Laere (BVVL) Syndrome and Other Bulbar SMA Syndromes

Brown-Vialetto-Van Laere syndrome is a rare autosomal recessive neurologic disorder of pontobulbar motor neurons (VII, IX, X, XI, and XII) with progressive facial weakness, dysphagia, tongue amyotrophy (Figure 2(e)), fasciculations, and sensorineural deafness in childhood [74, 75]. Hearing loss usually precedes the onset of the neurological syndrome which also includes optic atrophy, weakness of the arms and hands (Figure 2(f)), termed “child in the barrel,” and ataxia. The disease course is progressive yet variable. Respiratory failure may ensue over 6–18 months in early-onset cases, leading to recurrent chest infections and death. Survival to the 6th decade is reported, and more recently, adult rapid onset pontobulbar palsy has been recognised [76, 77].

Electrophysiology demonstrates absent brainstem auditory responses and a sensorimotor neur(on)opathy [78]. There is elevation of serum medium-chain acylcarnitines and deficient flavin levels in some patients. BVVL is caused by homozygous or compound heterozygous mutations in *SLC52A2*, *SLC52A3*, or heterozygous form in *UBQLN1* genes [74, 79, 80]. The *SLC52A2* and *SLC52A3* genes encode the riboflavin transporters RFVT2 and RFVT3 and variable expression is found in the small intestine, fetal brain, and spinal cord. Neuronal riboflavin deficiency is postulated to underlie the BVVL phenotype, with riboflavin critical in carbohydrates, amino acids, and lipid metabolism. In *SLC52A2* mutations, a sensorimotor neuropathy and optic atrophy are other common features [79].

Importantly, the genetic underpinnings of BVVL have provided a targeted therapeutic strategy with oral or intravenous high dose riboflavin supplementation with the customary dose 10 mg/kg/day. Clinical response may be variable, ranging from rapid improvement in motor function; strength, hearing, vision, weaning, or cessation of respiratory support; and normalisation of neurophysiological parameters over days to gradual improvement over 12 months, stabilization of clinical state, or rarely no response is observed [75, 81]. All abnormal acylcarnitine profiles and flavin levels normalised after riboflavin supplementation.

Other inherited bulbar motor syndromes include Fazio-Londe syndrome, which is similar to BVVL but with no hearing loss. The aetiology is secondary to *SLC52A3* pathogenic variants. Separately, Worster-Drought syndrome is a congenital nonprogressive suprabulbar paresis associated with X-linked or autosomal inheritance. No gene loci have yet been identified. Perisylvian polymicrogyria may be identified on neuroimaging. While bulbospinal muscular atrophy (BSMA or Kennedy's disease) is typically an adult disorder, occasional onset in adolescence may occur with muscle cramps, chewing difficulties, and abnormal sexual development [82]. The later hallmarks of Kennedy's disease include bulbar, proximal limb weakness, widespread and prominent fasciculations, loss of sensory function in the distal extremities, and androgen insensitivity. BSMA is an X-linked recessive neurodegenerative disorder caused by increased CAG repeats in the androgen receptor. The principal cellular disturbance caused by the mutant androgen receptor AR is attributed to transcriptional dysregulation, with secondary changes occurring in cell signalling, mitochondrial activity, and axonal transport.

## 4. Paediatric Multisystem Disorders Including Motor Neuron Disease

Motor neuron degeneration may be an important feature and morbidity of diseases under the “umbrella” of neurodegenerative or multisystem disorders. These are summarised in Table 2, with several described below and selected due to greater incidence, while remaining rare diseases. Meticulous clinical, electrophysiological, and pathological assessments reveal progressive weakness, amyotrophy, and denervation, even though these may not be the principal features, serving to accurately characterise phenotypes and guide management.

### 4.1. Infantile Neuroaxonal Dystrophy (INAD)

**Figure 3:**
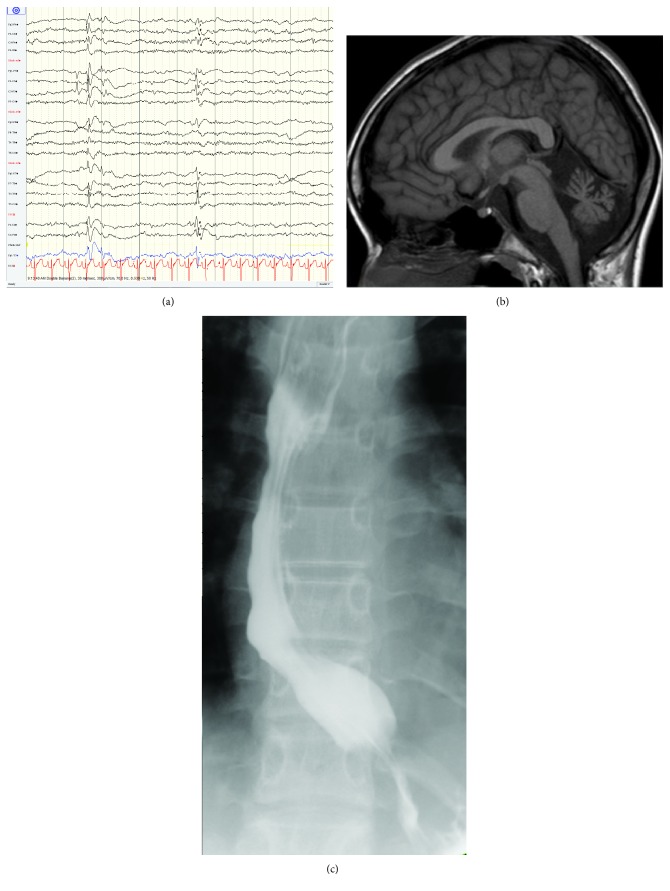
Clinical pictures of paediatric multisystem disorders with motor neuron disease. (a) Interictal EEG showing diffuse spike and slow wave complexes in infantile neuroaxonal dystrophy (INAD). (b) MRI brain in the sagittal plane showing cerebellar atrophy seen in INAD. (c) Barium swallow demonstrating dilated oesophagus and tapering in keeping with achalasia in AAA syndrome.

Classic infantile neuroaxonal dystrophy (INAD) is a severe neurodegenerative disease, typically with onset before age 3 years. It is initially characterised by strabismus, hypotonia, and developmental delay such that SMA variants may be considered among differential diagnoses, supported by weakness and denervation. Subsequently, psychomotor regression, spastic tetraplegia and extensor planar responses, dystonia, lower limb hyporeflexia, and autonomic dysfunction evolve. Ophthalmological findings also comprise nystagmus, incoordinate eye movements, and optic atrophy with decrease of visual acuity [83–85]. Eventually, muscle weakness produces feeding and respiratory difficulties, resulting in aspiration and pneumonia such that most patients die before age 10 years [86]. Seizures are not common, but when present, interictal EEG may show fast activity and bursts of diffuse spike and wave complexes (Figure 3(a)). Typical EEG during a seizure can consist of diffused, irregularly sharp, and high-voltage slow wave complexes with desynchronization afterwards [85, 87]. The pathological hallmark is axonal spheroids, tubulovesicular structures, and mitochondrial abnormalities in central and peripheral nerves [88]. These may be identified within myelinated and unmyelinated intramuscular nerves or peripheral nerve endings of the skin and conjunctivae. Degeneration also involves the corticospinal tract. Marked cerebellar atrophy has been observed in nearly all *PLAG26* patients on neuroimaging [83, 89] (Figure 3(b)), with insidious atrophy of the inferior cerebellar vermis, presence of T2-weighted hypersignal of the cerebellar cortex and hypointensity of the globus pallidus and substantia nigra (detected in later stages and explained by iron deposition). MRI spectroscopy also provides some important clues to diagnosis where studies have identified N-acetylaspartate reduction and the presence of lactate in the basal ganglia [85].

Homozygous mutations in *PLA2G6* are associated with a spectrum of neurodegenerative phenotypes: INAD, atypical neuroaxonal dystrophy, and *PLA2G6*-related dystonia and Parkinsonism. The *PLA2G6* gene maps on the long arm of chromosome 22q12.3-13.2, encoding a calcium-independent phospholipase A2 enzyme. It localises to the mitochondria and the enzyme is crucial for maintenance of cell membrane homeostasis, signal transduction, calcium signalling, cell proliferation, and apoptosis [90]. A novel gene in children presenting with distal motor neuropathy, spastic ataxia, and juvenile-onset brain iron accumulation has recently been identified and caused by biallelic mutations in *TBCE*, important in microtubule polymerization at the Golgi apparatus and its impairment leads to Golgi fragmentation previously seen in degenerating motor neurons in ALS [91].

### 4.2. AAA Syndrome

Achalasia-Addisonianism-Alacrima (AAA, triple A, or Allgrove syndrome) was first described in 1978. Alacrima is the earliest and most consistent feature and other ophthalmological findings may include optic atrophy or pallor, high astigmatism, and anisocoria [92]. Additional multisystem features include achalasia (Figure 3(c)), gastroparesis, glossitis, adrenal insufficiency, hyperpigmentation, palmar-plantar hyperkeratosis, short stature, osteoporosis, and microcephaly. Neurological manifestations may be varied and progressive and may include an ALS-like disorder, peripheral sensorimotor axonal neuropathy, autonomic dysfunction, spasticity, spinocerebellar syndrome, seizures (possibly related to hypoglycemia), mental retardation, or dementia, with onset during childhood or adulthood [93–95]. Related to the motor neuron disorder, bulbar manifestations may include dysarthria, dysphagia, tongue spasticity, and nasal speech. The increased tone, hyperreflexia, and abnormal central motor conduction time reflect cortical motor neuron dysfunction, although conventional MRI is normal [95].

Recessive mutations in the *AAAS* gene, encoding the Aladin or Adracalin protein, are associated with AAA syndrome. Chiefly, these are nonsense mutations, resulting in expression of a truncated protein. There is lack of genotype-phenotype correlation in AAAS, with interfamilial variability in the age of the onset of the disease described. The *AAAS* gene is ubiquitously expressed; however, particularly abundant expression is observed in the adrenal gland, gastrointestinal structures, pituitary gland, cerebellum, and fetal lung [96]. Function of Aladin is not well-understood, but the protein encoded belongs to the WD-repeat family of regulatory proteins and is part of the nuclear pore complex expressed in both neuroendocrine and cerebral structures. Nucleoporins are involved in transport between the nucleus and the cytoplasm, thus playing an important role in the cell cycle and gene expression of the peripheral and central nervous systems [97, 98].

### 4.3. Chédiak-Higashi Syndrome (CHS)

Chédiak-Higashi syndrome was first described in 1943 and is a rare, autosomal recessive systemic disease affecting the haematological and neurological systems, skin, and eyes. The former is characterised by hepatosplenomegaly, lymphadenopathy, pancytopaenia, and severe immunodeficiency with recurrent pyogenic infections and bleeding tendency. The accelerated phase presents with haemophagocytic lymphohistiocytosis. Partial oculocutaneous albinism may occur due to defects in pigmentation. Neurological involvement is variable, and motor neuronopathy accompanied by upgoing plantars has been described [99]. Cranial and peripheral neuropathy, cognitive impairment, seizures, ataxia, spinocerebellar degeneration, and extrapyramidal features such as Parkinsonism and dementia may also occur and are usually late-onset manifestations [100]. The pathognomonic feature of CHS is giant lysosomal vesicles within all cell types. MRI brain findings are nonspecific and cerebral or cerebellar atrophy may be seen and correlated with longer disease duration and more severe cognitive disabilities. Recently, morphologic variation of the posterior fossa was observed where the angle enclosing the superior cerebellar vermis was more acute in patients with CHS [101]. While bone marrow transplantation may alleviate the haematological and immunological complications and prolong survival beyond age 7 years, the neurological phenotype is not altered [102]. Levodopa may help with Parkinsonism. CHS is caused by homozygous mutations in the lysosomal tracking regulator gene, *CHS1/LYST* [103]. This is ubiquitously expressed and known to be involved in controlling the exocytosis of secretory lysosomes.

## 5. Juvenile Amyotrophic Lateral Sclerosis (jALS)

Amyotrophic lateral sclerosis (ALS) consists of progressive, although variable, degeneration of the corticospinal tract, brainstem, and spinal motor neurons with a worldwide incidence of 0.4–2.6 per 100,000 population [104]. It is now considered a multisystem disorder with various clinical presentations and predominant motor symptoms. The clinical course is also diverse and the natural history varies with age and sex. Approximately 90% of ALS cases are currently considered sporadic, related to complex polygenic and environmental factors, with a mean age of onset in the sixth decade, male-to-female ration of 1.7 : 1, and median survival from onset of symptoms of 2–4 years [105]. Together with variable penetrance genetic counselling is difficult. In contrast, hereditary ALS affects younger people, males and females equally, and has bimodal disease duration. Notably, juvenile ALS, with age of onset before 25 years, is more frequently familial than adult-onset forms and both autosomal dominant and recessive forms have been described (Table 3—ALS 2, 4, 5, 6, 6–21, and 16). Traditionally familial ALS with juvenile onset has been associated with slower progression and prolonged survival to encompass a normal life expectancy [106, 107]. More recently, distinct and apparently sporadic phenotypes have recently been recognised with aggressive presentations and course in adolescence attributable to specific mutations, for example, the fused-in-sarcoma gene (*FUS* or ALS6) or superoxide dismutase 1 (*SOD1*) [108]. Intriguingly, the pathology of juvenile ALS may also extend beyond the motor system, with emotional lability and inappropriate crying or laughing (pseudobulbar signs) apparent, similar to the ALS-FTD spectrum of disorders in adults, supporting the notion of multisystem neurodegenerative disorders and common pathomechanisms.

### 5.1. Amyotrophic Lateral Sclerosis 2 (ALS2)

ALS2 is characterised by childhood onset, mean 6.5 years, and progressive spasticity of facial and limb muscles with bulbar or pseudobulbar symptoms. The latter may include uncontrolled laughter and/or weeping and spastic dysarthria. Moderate muscle atrophy and cognitive impairment may also be evident [107]. ALS2 is very slowly progressive and life span may be normal, with only some individuals losing ambulation between ages 12 and 50 years. Variable phenotypes associated with younger age of onset, generalised dystonia and rapid progression, spastic quadriparesis, microcephaly, and cerebellar signs have been recognised [109, 110].

Recessive pathogenic mutations of the *Alsin* gene, chromosome 2q33, cause ALS2. *Alsin* encodes two protein transcripts by alternate splicing, a long form that includes three putative guanine exchange factor (GEF) domains and a short form. Most mutations cause truncation of long and/or short form of alsin protein with consequent loss of function. Alsin is produced in many tissues but in the highest amounts in the brain, especially motor neurons. The deleted mutation in the long form causes juvenile primary lateral sclerosis while the mutation that affects both long and short form causes ALS2 [111]. GEFs activate members of the Ras super family of GTPases to play a role in vesicle transport and membrane trafficking processes. Even though diverse functions of alsin have emerged, very little is known about its role in upper motor neurons. The phenotype of ALS2 also includes infantile onset ascending hereditary spastic paraplegia and juvenile primary lateral sclerosis [111, 112].

### 5.2. Juvenile-Onset Motor Neuron Disease with Dominant Inheritance and No Bulbar Involvement (ALS4)

ALS4 is characterised by juvenile onset, often before age 25 years (mean age of onset is 17 years), with slow progression, severe distal muscle weakness, pyramidal signs and preservation of cognition, and bulbar and sensory function. Initial symptoms are typically gait difficulties due to lower limb involvement and life expectancy may be normal [106, 113].

Autosomal dominant inheritance with mutations mapped to chromosome 9p34 in the senataxin (*SETX*) gene causes ALS4. The protein senataxin has DNA and RNA helicase activity, and it plays a role in DNA and RNA cellular regulation, with disruption leading to neurodegeneration [114]. *SETX* mutations are allelic with ataxia and oculomotor apraxia type 2 and have also been described in spinocerebellar ataxia 1, with autosomal recessive inheritance [115].

### 5.3. Juvenile Amyotrophic Lateral Sclerosis 5 (ALS5)

ALS5, closely resembling ALS1, accounts for 40% of autosomal recessive jALS and is characterised by a slowly progressive lower motor neuronopathy associated with spasticity. Onset is between the first and second decade (mean 16.3 years) with initial distal weakness and atrophy in the hands and feet followed ultimately by bulbar dysfunction [116, 117]. Progressive upper motor neuron disease becomes more obvious with time and most patients become wheelchair bound with disease duration of 20–40 years (mean of 34.3 years). No sensory or cognition changes and no thinning of corpus callosum are noted in these patients.

ALS5 is autosomal recessively inherited and has been linked to a 6 cM region of chromosome 15q15.1–q21.1 with both compound heterozygous and homozygous mutations described in the *spatacsin* gene [116]. This gene is ubiquitously expressed in the nervous system, predominantly in the cerebellum, cerebral cortex, hippocampus, and pineal gland. The spatacsin protein has a role in neuronal axonal maintenance including intracellular cargo trafficking. This gene was first described in spastic paraplegia 11 with thinning of corpus callosum (SPG11) and L-dopa-responsive Parkinsonism and recently found in Charcot-Marie-Tooth disease, axonal, and type 2X [118, 119].

### 5.4. Amyotrophic Lateral Sclerosis 6 with or without Frontotemporal Dementia (ALS6)

Early-onset, aggressive jALS presentations and rapid disease course, with progressive spastic gait, dysarthria, facial muscle involvement, and lower motor neuron dysfunction, are associated with mutations in the fused-in-sarcoma gene (*FUS* or ALS6). With up to 5% of familial ALS attributable to *FUS* gene mutations, dominant or recessive inheritance, and identification in individuals with sporadic ALS [108, 120] and ALS with Parkinsonism or frontotemporal dementia, *FUS* has emerged as a significant genetic factor in the ALS landscape. Consistent *FUS* genotype-phenotype correlations have been identified; for instance, p.S96del mutations are associated with longer survival, contrasting with a severe clinical course with death or permanent ventilation 15.4 months after onset in p.P525L mutations [108, 121, 122]. Separately, initial symmetric weakness of proximal limb muscles and neck flexor/extensors and minimal upper motor neuron involvement are associated with the R521C mutation [123]. While most patients have normal cognition, intellectual disability and learning difficulties may be present [121, 122, 124].

The pathological hallmark of *FUS*-associated MND is basophilic inclusions in motor neurons [125, 126]. *FUS* is ubiquitously expressed, predominantly nuclear, a DNA/RNA-binding protein that forms cytoplasmic aggregates, and is imported into the nucleus by transportin; it functions in DNA and RNA metabolism and is considered a proteinopathy.

### 5.5. Juvenile Amyotrophic Lateral Sclerosis 6-21 (ALS6-21)

A single consanguineous family, with four of six affected children, has been reported and designated ALS6-21, presenting between ages 4 and 10 years with prominent upper motor neuron features, marked distal muscle wasting of upper and lower limbs, and bulbar dysfunction. In addition, asymmetric ptosis, moderate facial weakness, mild sensory loss, and gynaecomastia were noted, with slow progression and loss of ambulation after a decade. Autosomal recessive inheritance with foci on chromosome 6p25 and 21q22 were found [127].

### 5.6. Juvenile Amyotrophic Lateral Sclerosis 16 (JALS16)

JALS16 relates to the description of a single consanguineous Saudi Arabian family that included six affected individuals with a slowly progressive lower limb spasticity and weakness and onset between ages 1 and 2 years. Upper limb involvement and loss of ambulation occurred by 20 years, with normal respiratory, bulbar, cognitive, sensory, and sphincter functions [128].

Recessive mutations in the sigma-1 receptor gene, *SIGMAR1*, causes JALS16. It is an endoplasmic reticulum chaperone binding to a wide range of ligands and also functions in the regulation of ion channels like potassium channel and could modulate neurotransmitter release and calcium transport in the mitochondria. It is thought to cause motor neuron pathology by altering endoplasmic reticulum morphology, lipid raft destabilization, and defective endolysosomal pathways [129]. It is also necessary for proper mitochondrial axonal transport in motor neurons; in particular, the retrograde movement of mitochondria and the mutant SIGMAR1 protein reduces mitochondrial ATP production, inhibits proteasome activity, and causes mitochondrial injury, aggravating endoplasmic reticulum stress-induced neuronal death [130]. While *SIGMAR1* is ubiquitously expressed, it is enriched in motor neurons of the brainstem and spinal cord with subcellular postsynaptic increases [131]. Recessive *SIGMAR1* mutations may also cause distal SMA2 and frontotemporal lobar degeneration [132, 133]. Furthermore, polymorphism of *SIGMAR1* gene is also implicated in Alzheimer's disease, schizophrenia, stroke, amnesia, and depression [134].

### 5.7. Other Genetic Causes of Juvenile ALS

As may be expected, the genes associated with jALS continue to increase and phenotypes broaden among known ALS genes and other neurodegenerative disorders [20, 25]. Mutations in *Ubiquilin 2* have been identified in X-linked dominant juvenile- and adult-onset ALS and ALSFTD [135]. The pathology is characterised by ubiquitinated inclusions that may be colocalised with the proteins TDP-42 and FUS. Furthermore, diagnostic review in cases with slow progression and prolonged survival may assist in focusing genetic testing. For example, repeat neuroimaging in a patient with jALS identified basal ganglia iron deposition, directing sequencing of genes associated with neurodegeneration with brain accumulation (NBIA) and confirming compound heterozygous mutation of the *C19ORF12* gene which encodes a transmembrane mitochondrial protein [136].

### 5.8. Treatment of jALS

Juvenile ALS remains without a curative treatment; however, multidisciplinary specialised clinics have increased survival and quality of life for adult patients with a focus on supportive care [137, 138]. Of possible therapeutic relevance, cortical hyperexcitability, a pathophysiological driver of adult ALS and partly ameliorated by riluzole (the only disease-modifying therapy in ALS), has not been assessed in juvenile ALS phenotypes [139, 140]. The advances in understanding ALS pathophysiology continue to reveal novel therapeutic targets, with gene therapies using antisense oligonucleotides demonstrating efficacy in preclinical models and entering phase 1 clinical trials for specific genetic subtypes of ALS [141, 142]. Accordingly, identifying the genetic factors that may disrupt molecular processes in juvenile ALS is of increasing therapeutic relevance.

## 6. Genetic Insights into Pathophysiology

**Figure 4:**
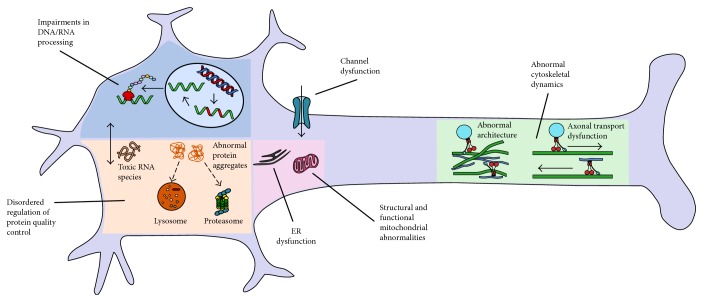
Pathogenic mechanisms underlying motor neuron disease in children of these genetic variants associated with paediatric motor neuron diseases relate to disordered regulation of autophagy/protein quality control (*ASAH1*, *UBE1*,*UBQLN1*, *LYST*, *ATXN3*, and *SCP2*), RNA processing (*VRK1*, *EXOSC3*, *EXOSC8*, *TSEN54*, *SLC254A6*, *MORC2*, *SMN1*, *TRIP4*, *ASCC1*, *UBA1*, *GLE1*, *ERBB3*, *IGHMBP2*, and *RBM28*), and cytoskeletal dynamics (*ASAH1*, *BICD2*, and *DYNC1H1*). Functional connections between pathways occur. Additional mechanisms include structural and functional abnormalities of mitochondria (*SOC2*, *TK2*, *DGUOK*, and *PLAG26*), molecular transport by cation channeling (*TRPV4* and *SCN1A*), and vitamin uptake (*SLC52A3* and *SLC52A2*).

The pathogenic mechanisms underlying motor neuron disorders in children and young people reverberate with the current understanding of ALS, including disordered regulation of autophagy/protein quality control, cytoskeletal dynamics, and RNA metabolism (Tables 1–3 and Figure 4) [143]. Functional connections between the main pathways are emerging, for instance altered autophagy is connected to impaired RNA metabolism and protein homeostasis, removing cytosolic components such as aggregates of proteins or damaged organelles. Ubiquitinated protein inclusions are common neuropathological features, further linking pathological processes. Additional mechanisms include structural and functional abnormalities of mitochondria, free radical-mediated oxidative stress, molecular transport by cation channeling (TRPV4), vitamin uptake (SLC52A3 and SLC52A2), nuclear transport (GLE1), lipid metabolism (ASAH1), and axonal transport (BICD2 and DYNC1H1).

## 7. Advances in Next Generation Sequencing and Clinical Application in Paediatric Motor Neuron Disorders

Paediatric motor neuron diseases are often dominantly or recessively inherited, such that diagnostic genetic testing is usually undertaken. The first line of investigation for a child or young adult patient suspected to have proximal SMA should be MLPA testing for homozygous deletion of exons 7 and 8 in the *SMN1* gene. Beyond SMN-related disease, paediatric motor neuron diseases display genetic and phenotypic heterogeneity, making diagnosis based on clinical examination or histological appearances alone difficult. Consequently, sequential gene testing may engender an expensive, long, and unfulfilling diagnostic odyssey. Even so, establishing the molecular genetic diagnosis is important in understanding the prognosis, accurate genetic counselling, enabling access to specific therapies and clinical trials. With the advent of NGS, the diagnostic paradigm is being revolutionised in genetic disorders, with the capability to capture and sequence a selection of genes, the entire exome, or genome. These innovative technologies have demonstrated efficacy in several patient cohorts with neuromuscular disorders, improving diagnostic rates, shortening the time and cost of diagnosis, and expanding phenotypes [144, 145]. In addition, NGS serves as a powerful tool to discover new pathways of disease, understand shared pathways in disease, and identify new targets for treatment. The AANEM considers performing genetic testing in neuromuscular patients is important in providing high-quality healthcare to neuromuscular patients, with clinical, safety, psychosocial, and research benefits, encouraging an update of clinical practice [146]. Of note, this approach differs to adult MND; recent European guidelines recommended genetic testing in patients with first- or second-degree relatives with MND or FTD with informed consent and counselling [147]. The latter includes understanding potential uncertainties about the pathogenicity and penetrance of some genetic mutations and deficient genotype-phenotype correlations. As NGS technologies are increasingly being used as a first-tier genetic test in the clinical realm, accurate clinical evaluation continues to be important. As such, an understanding of NGS and its application for paediatric patients with suspected genetic motor neuron disorders are imperative and summarised below.

NGS encompasses targeted panels, WES, and WGS. Targeted NGS, incorporating a panel of known genes, is commercially available and valuable in diagnosis in conditions with genetic locus heterogeneity and clinical variability [148]. Gene panels are a quick, relatively cost-effective and comprehensive disease diagnostic or rule-out test for certain conditions. WES analyses the protein's coding region, in which an estimated 85% of disease-causing mutations are believed to occur. The limitations of current targeted panels and WES include that they cannot reliably detect copy number variants and triple repeat changes. For example, SMN1 copy number and repeat sequences will be missed leading to false-negative results. Future iterations to improve the efficacy of targeted NGS approaches may incorporate copy number analysis. Further challenges include a large number of variant interpretation and reporting and variant of uncertain significance (VOUS). It is difficult to determine between individuals and rare and nonpathogenic variants versus disease-causing variants; therefore, there are validation tests that are vital to this such as in silico predictions, functional studies, complex segregation analysis in other family members, and absence of the mutation in controlled population. Even with these tools, there could still be too many variants to investigate further, especially when working with small families or isolated probands. These diseases include those which are so rare that the identification of additional families worldwide is required. The false-positive rate for NGS, especially for insertions and deletions, is still very high. Sanger sequencing is inevitable and necessary to remove the false-positive detections but increases cost and turnaround time. Standard pipelines are in place to process the sequencing data generated by WES, and they can be compared with various public databases such as the single-nucleotide polymorphism (SNP) database (dbSNP), the 1000 Genomes Project, the Exome Variant Server, and the International HapMap Project, as well as internal control databases to remove common variants and aid in identifying causative genes. A major limitation to WES is that pathogenic mutations can occur outside the exons or be due to small copy number variants which will only be amenable to identification through WGS. It is increasingly being used in undiagnosed patients with no detectable genome-wide gene-coding mutation. WGS allows examination of single-nucleotide variants, indels, structural variants, and copy number variants in both the 1% part of the genome that encodes protein sequences and the 99% of remaining noncoding sequences. WGS probes can also include mitochondrial genome probes. A major difficulty to WGS is that it will identify many more sequence polymorphisms that have no connection to disease. Currently, the means to filter the variants are limited and still undergoing development.

## 8. Conclusions

Genetic causes are common and diverse among paediatric motor neuron disease with various modes of inheritance patterns suggesting genomic testing (panel/exome/genome) as first-line investigations if available. Establishing the molecular genetic diagnosis is important in understanding the prognosis, enabling access to specific therapies and clinical trials, supporting accurate genetic counseling for future pregnancies, and allowing cessation of further invasive testing in an attempt for a diagnosis.

Clinical and genetic phenotypes of motor neuron disease include a wide spectrum, creating complexities in molecular genetic diagnosis, but with the arrival of NGS, the diagnostic paradigm has shifted and these innovative technologies have demonstrated efficacy in improving diagnostic rates, shortening the time and cost of diagnosis, and expanding phenotypes. Therefore, the recognition of currently known phenotypes and the genes associated is important for clinicians as we move onto utilising NGS in our diagnostic approach.

## Figures and Tables

**Table 1:** Spinal muscular atrophy plus syndromes with known gene variants.

Spinal muscular atrophy plus syndrome	Age of onset	Clinical phenotype	Gene	Gene function	Mode of inheritance	References
*Pontocerebellar hypoplasia with spinal muscular atrophy (PCH1)*	Early infancy	Severe hypotonia, areflexia, muscle weakness, central visual impairment, dysphagia, respiratory insufficiency, and acquired microcephaly. MRI brain shows cerebellar hypoplasia with variable involvement of the pons.	*VRK1* *EXOSC3* *EXOSC8* *TSEN54* *SLC254A6*	RNA processing	AR	[13, 15, 17–19, 149]
			*MORC2*	Mitochondrial DNA repair, RNA processing, lipid metabolism		
*Spinal muscular atrophy with progressive myoclonic epilepsy (SMAPME)*	Childhood (initial development normal)	Proximal muscle weakness, hypotonia, areflexia, and muscle wasting. Tongue fasciculation and may have sensorineural hearing loss, polyarticular arthritis, and facial weakness. Later myoclonic epilepsy	*ASAH1*	Cytoskeletal architecture—axonal branching Autophagy (lysosomes)	AR	[24, 25]
*SMA with skeletal abnormalities*						
SMA with congenital arthrogryposis and fractures	Antenatal	Arthrogryposis, fractures, cardiac defects, severe hypotonia, weakness, areflexia with tongue fasciculation, and respiratory insufficiency. Early death	*SMN1* *TRIP4* *ASCC1* *UBA1*	RNA processing	AR	[33, 34]
Spinal muscular atrophy, X-linked (SMAX2)	Antenatal	Congenital fractures, arthrogryposis, and tongue fasciculation	*UBE1*	Protein degradation via proteasomes	X-linked	
Lethal arthrogryposis with anterior horn cell disease (LAAHD)	Antenatal	Fetal akinesia deformation sequence. Death in utero or within days of delivery. Normal spinal cord. Finnish	*GLE1*	RNA processing—mRNA export mediator	AR	[40]
Lethal congenital contracture syndrome 1 (LCCS1)	Antenatal	Lethal in the fetal period, most severe form of arthrogryposis. Abnormal spinal cord with marked thinning. Finnish.	*GLE1*	RNA processing—mRNA export mediator	AR	[40]
Lethal congenital contracture syndrome 2 (LCCS2)	Antenatal	Congenital contractures, dysmorphism, and urinary bladder involvement. Majority early death. Israeli-Bedouin	*ERBB3*	RNA processing-modulator of phosphatidylinositol-3-kinase/Akt pathway	AR	[44]
*SMA plus disorders—mitochondrial related*						
Cardioencephalomyopathy with cytochrome C oxidase deficiency (CEMCOX1)	Infancy	SMA phenotype with hypertrophic cardiomyopathy, seizures, psychomotor retardation, and ophthalmoplegia. MRI brain—white matter and basal ganglia abnormalities	*SCO2*	Mitochondrial structure and function	AR	[45]
Mitochondrial depletion syndrome 2 (MTDP2)	Infancy/childhood	Hypotonic muscle weakness, respiratory failure, psychomotor retardation with seizures and ophthalmic involvement. Progressive and wide variability	*TK2*	Mitochondrial function—depletion of mitochondrial DNA	AR	[48]
Mitochondrial depletion syndrome 3 (MTDP3)	Infancy	SMA with infantile onset liver dysfunction, nystagmus, cerebral atrophy, and early death	*DGUOK*	Mitochondrial function—depletion of mitochondrial DNA	AR	[47]
*SMA plus with distinctive patterns of weakness*						
Spinal muscular atrophy, lower extremity predominant 1 (SMALED1)	Congenital to adult	Nonprogressive, proximal > distal leg only weakness MRI lower limb—sparing of thigh adductors and semitendinosus	*DYNC1H1*	Cytoskeletal dynamics—dynein complex for axonal transport	AD	[53]
Spinal muscular atrophy, lower extremity predominant 2 (SMALED2)	Congenital to adult	Slow progression, proximal > distal leg > arms weakness with some contractures	*BICD2*	Cytoskeletal dynamics dynein-dynactin complex	AD	[54, 55]
Scapuloperoneal spinal muscular atrophy (SPSMA)	Early adult	Progressive weakness of face and pectoral muscles with laryngeal palsy. May have sensorineural deafness, skeletal abnormalities. MRI lower limb—sparing of biceps femoris and medial gastrocnemius	*TRPV4*	Calcium channel	AD	[62]
Congenital distal spinal muscular atrophy (DSMA)	Congenital	Nonprogressive proximal and distal legs only weakness and contractures	*TRPV4*	Calcium channel	AD	[62]
Spinal muscular atrophy with respiratory distress (SMARD)	Infancy	Distal > proximal lower limb > upper limb weakness with early diaphragm weakness and respiratory insufficiency	*IGHMBP2*	Ribosomal biogenesis in RNA processing	AR	[72, 73]
Spinal muscular atrophy with respiratory distress 2 (SMARD2)			*LASIL*		X-linked	[64]
Brown-Vialetto-Van Laere (BVVL) syndrome	Early childhood to adulthood	Progressive pontobulbar palsy with weakness of the arms, hands, and face; ataxia; dysphagia; tongue wasting; and fasciculations and sensorineural deafness	*SLC52A3 SLC52A2 UBQLN1*	Vitamin transport Protein degradation through proteasome	AR	[74, 79, 80]

SMA plus syndromes, or atypical SMA, encompass disorders in which lower motor neuron dysfunction is the primary but not the sole feature and may be ordered by distinct patterns of muscle weakness. AD: autosomal dominant; AR: autosomal recessive; RNA: ribonucleic acid.

**Table 2:** Paediatric multisystem disorders with motor neuron disease.

Spinal muscular atrophy plus syndrome	Age of onset	Clinical phenotype	Gene	Gene function	Mode of inheritance	References
Infantile neuroaxonal dystrophy (INAD)	Early childhood	Initial strabismus, hypotonia, weakness, and developmental delay. Subsequent psychomotor regression, spastic tetraplegia, dystonia, nystagmus, optic atrophy	*PLA2G6*	Mitochondrial structure and function	AR	[90]
Allgrove syndrome (AAAS)	Childhood to adulthood	Adrenal insufficiency, Alacrima, achalasia. May develop an ALS-like disorder, peripheral sensorimotor axonal neuropathy, autonomic dysfunction, spasticity, spinocerebellar syndrome, seizures, mental retardation or dementia +/−palmar-plantar hyperkeratosis, microcephaly	*AAAS*	Regulatory protein	AR	[98]
Chédiak-Higashi syndrome (CHS)	Early to adult	Polyneuropathy, mental retardation, hypopigmentation, photophobia, and systemic disease (immunologic defects, bleeding diathesis)	*LYST*	Autophagy—lysosomal trafficking regulator	AR	[100]
Distal SMA with encephalopathy	Early	Distal motor neuropathy, spastic ataxia, and juvenile-onset brain iron accumulation	*TBCE*	Microtubule polymerization at Golgi apparatus	AR	[91]
Alopecia, progressive neurological defects & endocrinopathy (ANE)	2nd decade	Alopecia, severe mental retardation, progressive motor deterioration, central hypogonadotropic hypogonadism, central adrenal insufficiency, short stature, microcephaly, and hypodontia. MRI shows hypoplastic pituitary gland	*RBM28*	RNA processing—regulation of ribosome biogenesis	AR	[150]
Spinocerebellar ataxia type 3 (Machado-Joseph disease)	From the 2nd decade	Ataxia, facial, and lingual fasciculations and variable pyramidal disturbances, dystonia, Parkinsonism, sleep disorders, rigidity, peripheral neuropathy, distal muscle atrophy, or external ophthalmoplegia	*ATXN3 (larger expansions)*	Protein quality control—ubiquitin proteasome system	AD	[151]
Leukoencephalopathy with dystonia & motor neuropathy	2nd–4th decade	Dystonia, leukoencephalopathy with cerebellar signs, motor neuropathy, pyramidal and posterior column abnormalities, and azoospermia	*SCP2*	Autophagy—peroxisomal enzyme	AR	[152]
Motor neuronopathy with cataracts and skeletal abnormalities	Early to adult	Distal arm and proximal leg weakness, cataracts, short stature, dysplastic skull base, and small carpal bones	*ALDH18A1*	Amino acid biosynthesis	AD	
Dravet syndrome with motor neuropathy	Infancy	Epileptic encephalopathy, ataxia, developmental delay, pes valgus. Later onset gait abnormality—may be crouched, motor neuropathy	*SCN1A*	Ion channel function	AR	[153]

Motor neuron degeneration may be an important feature and morbidity of diseases in neurodegenerative or multisystem disorders. Clinical, electrophysiological, and pathological assessments reveal progressive weakness, amyotrophy, and denervation, even though these may not be the principal features, serving to accurately characterise phenotypes and guide management. AD: autosomal dominant; AR: autosomal recessive; RNA: ribonucleic acid.

**Table 3:** Juvenile amyotrophic lateral sclerosis.

Spinal muscular atrophy plus syndrome	Age of onset	Clinical phenotype	Gene	Gene function	Mode of inheritance	References
Amyotrophic lateral sclerosis 2 (ALS2)	Childhood	Spasticity and weakness of facial and limb muscles with bulbar or pseudobulbar symptoms. May have cognitive impairment. Slowly progressive.	*Alsin*	GEF signalling	AR	[111]
Amyotrophic lateral sclerosis 4 (ALS4)	Young adult	Severe distal weakness and pyramidal signs. Preservation of bulbar function and cognition. Slow progression.	*Senataxin*	DNA repair and RNA processing	AD	[114]
Amyotrophic lateral sclerosis 5 (ALS5)	1st to 2nd decade	Progressive distal weakness and amyotrophy. Later spasticity and bulbar symptoms. Wheelchair bound in 20–40 years.	*Spatacsin*	Axonal maintenance	AR	[116]
Amyotrophic lateral sclerosis 6 (ALS6)	Mean 40 years but can be juvenile	Rapidly progressive spastic gait, weakness, amyotrophy, dysarthria, and facial muscle involvement +/− frontotemporal dementia or cognitive impairment	*FUS*	Transcription, RNA processing and DNA repair	AR or AD	[120]
Amyotrophic lateral sclerosis 6-21 (ALS6-21)	Childhood	Single family with prominent spasticity, upper and lower limb distal weakness, and bulbar dysfunction. Ptosis, facial weakness, and gynaecomastia	*Unknown*, 6p25, and 21q22	Uncertain	AR	
Amyotrophic lateral sclerosis 16 (ALS16)	Infancy or childhood	Single family in Saudi Arabia with lower limb spasticity and weakness then upper limb involvement.	*SIGMAR1*	Endoplasmic reticulum stress and mitochondrial dysfunction	AR	[128]

Juvenile amyotrophic lateral sclerosis (jALS) consists of progressive, although variable, degeneration of the corticospinal tract, brainstem, and spinal motor neurons with onset before 25 years. AD: autosomal dominant; AR: autosomal recessive; RNA: ribonucleic acid.
